# Circulating Irisin Level as a Biomarker for Pure Aortic Stenosis and Aortic Valve Calcification

**DOI:** 10.1007/s12265-022-10327-9

**Published:** 2022-10-12

**Authors:** Shan-shan Wang, Jia-min Li, Po Hu, Yu-chao Guo, Xian-bao Liu, Jian-an Wang, Han Chen

**Affiliations:** Department of Cardiology, Second Affiliated Hospital, Zhejiang Provincial Key Lab of Cardiovascular Disease Diagnosis and Treatment, Zhejiang University School of Medicine, 88 Jiefang Road, Hangzhou, 310009 Zhejiang China

**Keywords:** Irisin, Aortic stenosis, Calcification, Biomarker, Transcatheter aortic valve implantation

## Abstract

Irisin, a myokine mainly secreted by skeletal and cardiac muscles, is actively involved in cardiovascular diseases. However, whether irisin is associated with aortic stenosis remains unknown. Two hundred ninety-three severe AS patients who underwent transcatheter aortic valve implantation were enrolled and followed-up for 35 months on average. Enzyme-linked immunosorbent assay (ELISA) was applied to measure circulating irisin levels. Patients were divided into two groups based on the median plasma irisin level. We found that high plasma irisin levels were independently associated with pure aortic stenosis (PAS) after adjusting for age, body mass index, history of peripheral vascular disease, and creatinine (*OR* = 3.015, 95% *CI* 1.775–5.119, *P* < 0.001). ROC curve analysis showed a significant predictive value of irisin for PAS (*AUC* = 0.647, 95% *CI* 0.583–0.711, *P* < 0.001). The severity of aortic valve calcification was negatively associated with plasma irisin levels (*P* < 0.05). In conclusion, irisin is an independent predictor for PAS and is negatively associated with the severity of aortic valve calcification.

**Supplementary Information:**

The online version contains supplementary material available at 10.1007/s12265-022-10327-9.

## Introduction

Aortic stenosis (AS) is the most common valvular heart disease in elderly people. The prevalence of AS increases with age, accounting for only 0.2% in people aged 50 to 59 years, and increasing to 9.8% in people aged ≥ 80 years [1]. Progressive narrowing of the aortic valve leads to an increased pressure gradient, left ventricular hypertrophy, and left ventricular diastolic dysfunction, which subsequently leads to a decrease in exercise tolerance and even heart failure. Studies have shown that patients with AS have a long asymptomatic period during which their survival rates are similar to those of healthy people. Once typical symptoms appear, such as dyspnea, syncope, and angina, survival rates are sharply reduced [2]. Patients with severe aortic stenosis with symptoms have an annual mortality rate of approximately 25%, and 75% of patients die within 3 years [3]. Current academic guidelines recommend intervention in patients with severe aortic stenosis who have symptoms, significantly reduced left ventricular ejection fraction (< 50%), or rapid progression [4]. Since its first clinical application in 2002, transcatheter aortic valve implantation (TAVI) has provided a new option for patients who cannot tolerate surgery or who are at high risk of surgery [5], and its efficacy has been fully recognized in multiple clinical studies. Thus, there is a trend to expand TAVI to patients with moderate- to low-risk of surgery. A systematic preoperative assessment of TAVI, including anatomy, hemodynamic status, and surgical risk assessment, is critical to determine the suitability and benefit of TAVI treatment in patients with AS. Currently available mainstream surgical risk scoring system includes the Society of Thoracic Surgeons (STS) score and the European System for Cardiac Operative Risk Evaluation (EuroSCORE) score. However, their association with the peri-operative risk of interventional operation was not as significant as that noted for surgery [6]. In addition, frailty (muscle weakness, muscle atrophy, malnutrition, and slow gait) and neurocognitive function are also major factors influencing TAVI postoperative outcomes [7], and these variables were incorporated into the preoperative risk assessment system for TAVI in patients with AS. The above assessment scoring system is computationally complex, especially for the assessment of frailty, which is susceptible to subjective factors. Thus, more objective and reliable biomarkers are needed to create an ideal tool for interventional risk assessment and prognosis analysis in patients with AS.

Irisin, a 112-amino acid myokine cleaved from the plasma membrane protein fibronectin type III domain containing protein 5 (FNDC5), is mainly secreted in response to exercise [8]. In addition to skeletal muscle, cardiac muscle can also secrete large amounts of irisin [9]. Previous studies demonstrated that this myokine-regulated metabolism, affected energy supply, and was associated with multiple metabolic diseases, such as diabetes mellitus (DM), chronic kidney disease, and macrovascular disease [10]. Some studies reported that plasma irisin levels were significantly reduced and negatively correlated with creatine phosphokinase-myocardial band isoenzyme (CK-MB) levels in infarct rats and patients with myocardial infarction (MI)[11–13]. Plasma irisin level could be applied to predict mortality in patients with acute heart failure (AHF)[14]. However, whether irisin is associated with aortic stenosis remains unknown. Reduced muscle mass is common in AS patients due to decreased activity tolerance. Studies have demonstrated that sarcopenia is strongly associated with prognosis after TAVI in AS patients [15]. Low levels of circulating irisin are a sensitive marker of muscle weakness and atrophy[16]. Combined with the strong association between frailty state and postoperative prognosis of AS patients after TAVI, we believe that plasma irisin levels may be easier to measure and quantify than frailty state or muscle content, and may also be related to AS patients prognosis after TAVI.

In the current study, a total of 293 severe AS patients who underwent TAVI were consecutively enrolled to explore the association between circulating irisin levels and baseline characteristics in AS patients and to assess whether baseline irisin levels could predict mortality in symptomatic AS patients who underwent TAVI.

## Methods

This study consecutively enrolled symptomatic AS patients who underwent TAVI in the Second Affiliated Hospital, Zhejiang University School of Medicine (SAHZU) from March 2013 to November 2018. The inclusion criteria were as follows: hospitalization for symptomatic AS, underwent TAVI, 18 years or older, severe AS (mean gradient evaluated by echocardiography). We excluded those who previously performed coronary artery bypass grafting surgery. Finally, 293 patients who completed the TAVI procedure were included in our study. Patients were followed up at 1 month and every year after the TAVI procedure. The last follow-up visit for the current study occurred in January 2020. The median follow-up period was 35 months. Baseline characteristics and echocardiographic and computed tomography (CT) parameters were collected before the TAVI procedure. We also obtained survival data on all-cause mortality, cardiovascular mortality, or noncardiovascular mortality (Valve Academic Research Consortium (VARC)- 2 definition) during the follow-up. The plasma irisin level was measured from blood samples obtained before the TAVI procedure using an enzyme-linked immunosorbent assay (ELISA) kit (Catalog No. EK-067–29, Phoenix Biotech, TX, USA), according to the manufacturer’s manual. The study complied with the principles of the Declaration of Helsinki regarding investigation in humans and this study protocol was approved by the Human Research Ethics Committee at SAHZU. All patients provided written informed consent.

For the present analysis, the patient population was divided into 2 groups based on the median pre-TAVI plasma irisin level reading (13.70 ng/mL). The primary study endpoint was overall survival after TAVI treatment. Cardiovascular death and noncardiovascular death were considered to be the secondary study endpoints. Death was confirmed by inspection of the death certificate or verified with a family member.

SPSS Statistics for Windows (IBM, NY, USA), version 25.0 was used to perform all statistical analyses. Continuous variables were tested for normality of distribution with the Shapiro–Wilk test and are presented as the mean ± SD or median and interquartile range (IQR). Differences between two groups of patients were analyzed using Student’s *t* test (normally distributed variables) or the Wilcoxon rank-sum test (nonnormally distributed variables). Categorical variables were expressed as numbers (percentage) and were compared using *χ*^2^ tests or Fisher’s exact test when appropriate. Spearman rank correlation analysis and logistic regression analysis were conducted to explore the associations of baseline variables and clinical outcomes with circulating irisin levels. Kaplan‒Meier analysis was used to evaluate and compare the time-to-event curve between the two groups. All analyses were considered statistically significant at a two-tailed *P* value of less than 0.05.

Variables were selected based on overall clinical relevance. Clinical characteristics including age, sex, body mass index (BMI), smoking status, frailty (KATZ score < 6) [17], Society of Thoracic Surgeons (STS) score, pure aortic stenosis (PAS) or mixed aortic valve disease (MAVD), NYHA class, medical history of dyslipidemia, DM, hypertension, stroke, MI, peripheral vascular disease (PVD), and previous percutaneous coronary intervention (PCI) were recorded. Serum or plasma creatinine levels, estimated glomerular filtration rate (eGFR), pro-brain natriuretic peptide (pro-BNP), CK-MB, and cardiac troponin-T (cTnT) were measured from venous blood samples prior to TAVI procedures. Echocardiography was performed in all patients to record the left ventricular ejection fraction (LVEF), left atrium size (LA), pulmonary artery systolic pressure (PASP), left ventricular end-diastolic dimension (LVEDd), mean pressure gradient across the aortic valve (mPG), aortic valve area (AVA), maximum transaortic velocity (V_max_), aortic regurgitation (AR) grade, mitral stenosis (MS) grade, mitral regurgitation (MR) grade, tricuspid regurgitation (TR) grade, and bicuspid or tricuspid aortic valve (BAV or TAV) at baseline. Aortic valve type (BAV versus TAV) and calcification grade were assessed by CT using 3mensio software (3mensio Medical Imaging BV, Bilthoven, the Netherlands). The AR/MS/MR/TR grade was categorized as follows: grade 0—none, 1—mild, 2—moderate, 3—moderate to severe, 4—severe. Aortic valve calcifications were graded in a 5-scale semiquantitative system: grade 0—noncalcification; grade 1—mild calcification (small isolated spots); grade 2—moderate calcification (multiple large spots); grade 3—severe calcification (extensive calcification of all cusps with fusion); and grade 4—massive calcification (large calcifications outreaching the annulus level) [18].

## Results

### Study Population

A total of 293 severe AS patients who underwent TAVI in SAHZU were included. The baseline clinical characteristics, laboratory findings, and echocardiographic or CT parameters of the study population are shown in Tables 1 and 2. The average age of the study population was 77 years and 58% were male. The median irisin level reading was 13.70 (IQR 10.95–17.96) ng/mL. The differences in age, frailty, CK-MB level, creatinine level, PAS, medical history of PVD, and dyslipidemia between the two groups all reached statistical significance (*P* < 0.05). Participants with high irisin levels were older and had relatively lower CK-MB levels, lower creatinine levels, and less frailty, PVD or dyslipidemia medical history. These patients also had a higher prevalence of PAS. Regarding echocardiographic features, a smaller LVEDd, lower PASP, and less aortic calcification were found, whereas a higher prevalence of BAV was observed in the high irisin level group (*P* < 0.05). Diverse aortic valve calcification grades and aortic regurgitation grades all reached significant differences between the two groups of AS patients in general (*P* < 0.05).

**Table 1:** Baseline clinical characteristics according to baseline irisin level

	Low irisin level (*n* = 147)	High irisin level (*n* = 146)	*P*-value
Age, yrs	76.00 (70.00,80.00)	78.00 (74.00,82.00)	0.002
Male	63.3% (93/147)	52.7% (77/146)	0.068
BMI, kg/m^2^	22.51 ± 3.31	22.73 ± 3.86	0.590
Smoker	11.6% (17/147)	13.7% (20/146)	0.582
Dyslipidemia	28.6% (42/147)	17.1% (25/146)	0.020
Diabetes mellitus	19.0% (28/147)	24.7% (36/146)	0.245
Hypertension	53.7% (79/147)	54.8% (80/146)	0.856
Frailty	93.9% (138/147)	55.5% (81/146)	< 0.001
NYHA class			0.067
ӀӀ	10.2% (15/147)	11.6% (17/146)	
ӀӀӀ	50.3% (74/147)	37.0% (54/146)	
ӀV	39.5% (58/147)	51.4% (75/146)	
PAS	54.4% (80/147)	77.4% (113/146)	< 0.001
Prior MI	2.7% (4/147)	0.7% (1/146)	0.371
Prior stroke	6.8% (10/147)	4.8% (7/146)	0.462
PVD	28.6% (42/147)	15.8% (23/146)	0.008
Prior PCI	12.2% (18/147)	13.0% (19/146)	0.843
Creatinine, μmol/L	82.00 (68.00,108.00)	76.50 (64.00,94.00)	0.026
eGFR, mL/(min × 1.73 m^2^)	71.16 (52.56, 89.61)	78.97 (57.11, 89.02)	0.274
Pro-BNP, pg/mL	3645.00 (1232.00, 8863.00)	2592.00 (935.00, 7426.00)	0.214
CK-MB, U/L	13.00 (10.00, 18.00)	12.00 (8.00, 14.25)	0.002
cTnT, ng/mL	0.026 (0.015, 0.048)	0.028 (0.017, 0.050)	0.443
STS score	5.50(3.90,8.83)	6.13 (4.02,9.35)	0.407

Dyslipidemia was defined as low-density lipoprotein cholesterol levels ≥ 160 mg/dL, high-density lipoprotein cholesterol levels < 40 mg/dL, triglycerides levels ≥ 150 mg/dL, or lipid-lowering medication use; Diabetes mellitus was defined as fasting blood glucose levels ≥ 126 mg/dL or anti-diabetic medication use; Hypertension was defined as systolic blood pressure ≥ 140 mmHg, diastolic blood pressure ≥ 90 mmHg, or anti-hypertensive medication use; Frailty was defined as KATZ score < 6

*NYHA* New York Heart Association, *PAS* pure aortic stenosis, *Prior MI* prior myocardial infarction, *PVD* peripheral vascular disease, *Prior PCI* prior percutaneous coronary intervention, *Pro-BNP* pro-B-type natriuretic peptide, *CK-MB* creatine phosphokinase-myocardial band isoenzyme, *cTnT* cardiac troponin T, *STS* Society of Thoracic Surgeons

**Table 2:** Baseline Echocardiographic and CT characteristics according to baseline irisin level

	Low irisin level (*n* = 147)	High irisin level (*n* = 146)	*P*-value
LVEF, %	55.00 (43.00, 62.40)	58.40 (44.93, 65.73)	0.068
LVEDd, cm	5.08 (4.51, 5.68)	4.76 (4.16, 5.34)	0.001
LA, cm	4.26 ± 0.69	4.20 ± 0.64	0.502
Mean gradient, mmHg	52.00 (42.00, 66.00)	54.00 (42.75, 67.25)	0.347
AVA, cm^2^	0.59 (0.45, 0.72)	0.56 (0.43, 0.73)	0.345
V_max_, ms	4.75 (4.25, 5.30)	4.80 (4.25, 5.40)	0.390
PASP, mmHg	42.00 (32.00, 57.50)	36.00 (30.00, 47.00)	0.008
AR			0.033
None (0)	13.6% (20/147)	21.2% (31/146)	
Mild (1)	44.2% (65/147)	51.4% (75/146)	
Moderate (2)	36.7% (54/147)	21.9% (32/146)	
Moderate to severe (3)	5.4% (8/147)	5.5% (8/146)	
MS			0.895
None (0)	95.2% (140/147)	96.6% (141/146)	
Mild (1)	3.4% (5/147)	2.1% (3/146)	
Moderate (2)	1.4% (2/147)	1.4% (2/146)	
MR			0.808
None (0)	15.6% (23/147)	12.3% (18/146)	
Mild (1)	61.2% (90/147)	63.7% (93/146)	
Moderate (2)	19.7% (29/147)	19.2% (28/146)	
Moderate to severe (3)	3.4% (5/147)	4.8% (7/146)	
TR			0.666
None (0)	34.0% (50/147)	32.2% (47/146)	
Mild (1)	48.3% (71/147)	54.1% (79/146)	
Moderate (2)	12.9% (19/147)	11.0% (16/146)	
Moderate to severe (3)	4.8% (7/147)	2.7% (4/146)	
Valve calcification	93.9% (138/147)	81.5% (119/146)	0.001
Calcification grade			
None (0)	6.1% (9/147)	17.8% (26/146)	
Mild (1)	9.5% (14/147)	10.3% (15/146)	< 0.001
Moderate (2)	19.7% (29/147)	26.0% (38/146)	
Severe (3)	32.0% (47/147)	33.6% (49/146)	
Massive (4)	32.7% (48/147)	12.3% (18/146)	
BAV	39.5% (58/147)	54.1% (79/146)	0.014

AR/MS/MR/TR: 0—none, 1—mild, 2—moderate, 3—moderate to severe, 4—severe; Calcification distribution: 0—symmetry, 1—asymmetry; Calcifications grade: 0—none calcification, 1—mild calcification (small isolated spots), 2—moderate calcification (multiple large spots), 3—severe calcification (extensive calcification of all cusps with fusion), 4—massive calcification (big calcification outreaching the annulus level)

*LVEF* left ventricular ejection fraction, *LVEDd* left ventricular end-diastolic dimension, *LA* left atrium, *AVA* aortic valve area, *V*_*max*_ max transaortic velocity, *PASP* pulmonary artery systolic pressure, *AR* aortic regurgitation, *MS* mitral stenosis, *MR* mitral regurgitation, *TR* tricuspid regurgitation, *BAV* bicuspid aortic valve

However, no significant disparities in sex, BMI, eGFR, pro-BNP, cTnT, smoking status, history of previous PCI, STS score, or NYHA class were noted between the two groups. In addition, there was no association between plasma irisin levels and comorbidities of severe AS patients, including DM, hypertension, stroke, or MI (*P* > 0.05 for all). Furthermore, baseline echocardiographic parameters including LVEF, LA size, AVA, mPG, V_max_, MS grade, MR grade, and TR grade were comparable between the two groups.

### Clinical Associations of Plasma Irisin Levels in AS

**Fig. 1:**
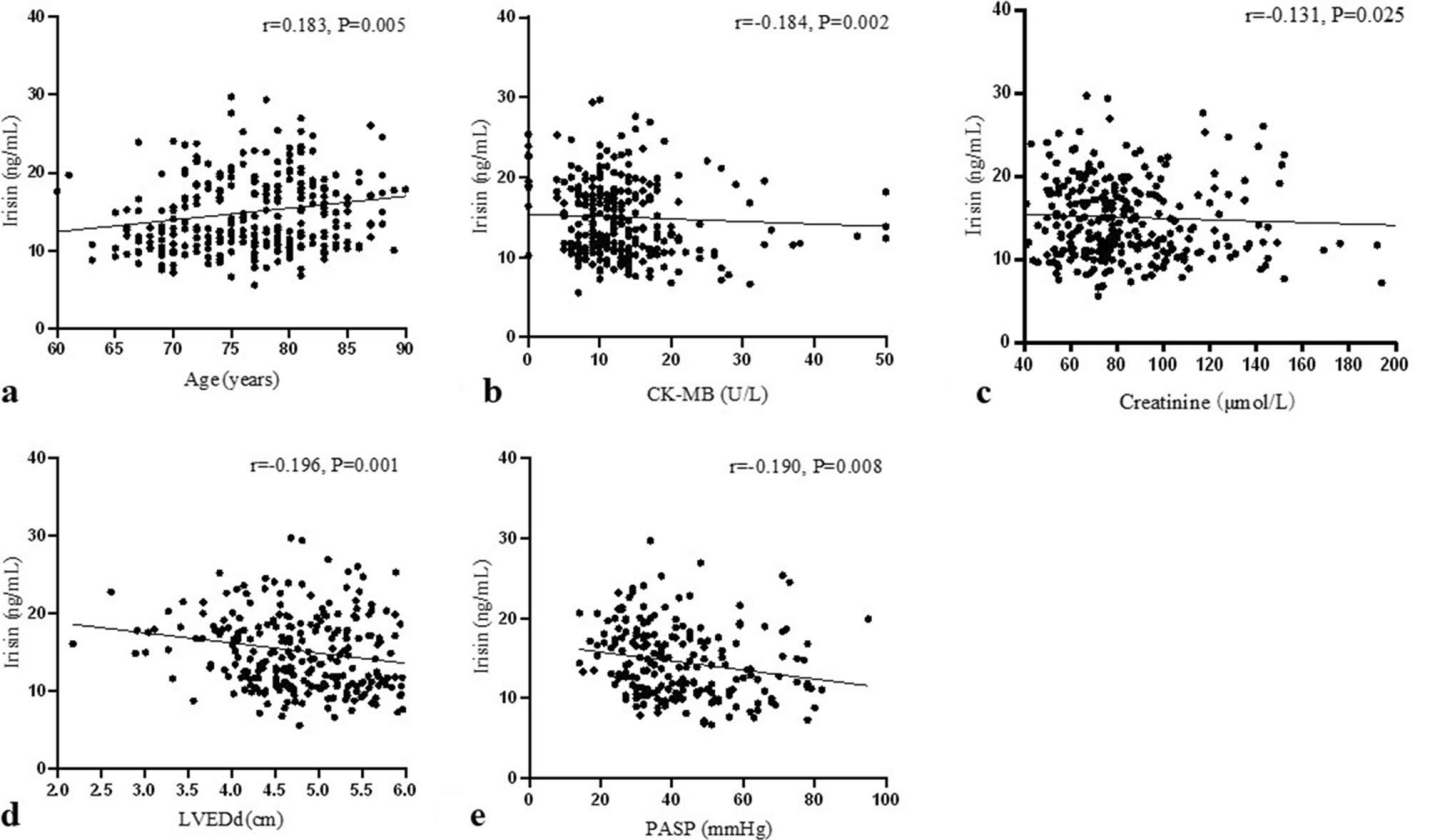
The scatter plots of correlation between plasma irisin levels and age (**a**), CK-MB (**b**), creatinine (**c**), LVEDd (**d**), PASP **(e**) in aortic stenosis patients

To further uncover the associations between baseline variables and irisin levels, Spearman rank correlation analysis analyses were performed (Table 3). Frailty, PAS or MAVD, and aortic valve calcification grade were strongly associated with plasma irisin levels (*P* < 0.001). In addition, age, creatinine, CK-MB, medical history of PVD, and dyslipidemia were also significantly different between the two irisin groups (*P* < 0.05). In addition, several imaging characteristics, such as LVEDd, PASP, BAV or TAV, and AR grade, also exhibited statistically significant differences in patients with different plasma irisin levels (*P* < 0.05). A scatter plot of the correlation between irisin and continuous variables is presented in Fig. 1.

**Table 3:** Spearman rank correlation analysis of clinical and imaging variables with irisin

Variables	Spearman *r*	*P*-value
Age, years	0.183	0.005
Creatinine, μmol/L	− 0.131	0.025
CK-MB, U/L	− 0.184	0.002
LVEDd, cm	− 0.196	0.001
PASP, mmHg	− 0.190	0.008
Frailty	0.537	< 0.001
Hyperlipidemia	− 0.136	0.020
PAS	0.242	< 0.001
PVD	− 0.154	0.008
AR	− 0.153	0.009
Valve calcification	− 0.188	0.001
Calcification grade	− 0.253	< 0.001
BAV	0.147	0.012

**Fig. 2:**
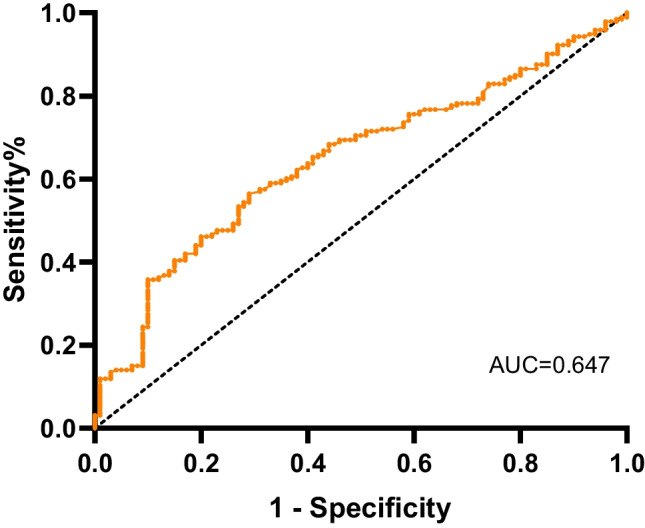
The plasma irisin level showed a strong predictive value for pure aortic stenosis (*AUC* = 0.647, 95% *CI* 0.583–0.711, *P* < 0.001)

Then we conducted logistic regression analysis to certify associations among frailty, PAS or MAVD, aortic valve calcification grade, and plasma irisin level. After adjusting for age, sex, medical history of DM, and creatinine level, a low plasma irisin level was independently associated with frailty (*OR* = 0.091, 95% *CI* 0.043–0.195, *P* < 0.001). In addition, multivariate logistic regression analysis revealed that a high plasma irisin level was independently associated with PAS when adjusting for age, BMI, medical history of PVD, and creatinine level (*OR* = 3.015, 95% *CI* 1.775–5.119, *P* < 0.001). Further receiver operating characteristic (ROC) curve analysis showed a significant predictive value of plasma irisin levels for PAS (*AUC* = 0.647, 95% *CI* 0.582–0.711, *P* < 0.001) (Fig. 2). The cutoff value of irisin for the diagnosis of PAS was 14.20 ng/mL. Univariate logistic regression analysis demonstrated that patients with severe and massive aortic valve calcification had significantly lower plasma irisin levels than patients with no aortic valve calcification (Table 4). After adjusting for age, sex, BMI, creatinine, and medical history of hyperlipidemia, hypertension, and DM, plasma irisin levels remained significantly different between patients with massive aortic valve calcification and no aortic valve calcification (*OR* = 0.158, 95% *CI* 0.060–0.415, *P* < 0.001).

**Table 4:** The association of plasma irisin levels with the prevalence of aortic valve calcification

Aortic valve calcification grade					
	None (35/293)	Mild (29/293)	Moderate (67/293)	Moderate (96/293)	Severe (66/293)
Unadjusted *OR*	1.000	0.371	0.454	0.361	0.130
[95% *CI*]	(Reference)	[0.130–1.061]	[0.185–1.114]	[0.153–0.850]	[0.051–0.330]
* P* value		0.064	0.085	0.020	< 0.001
Adjusted* *OR*	1.000	0.394	0.589	0.446	0.158
[95% CI]	(Reference)	[0.131–1.184]	[0.230–1.507]	[0.182–1.089]	[0.060–0.415]
* P* value		0.097	0.269	0.076	< 0.001

*CI* confidence interval,

^*^Adjusted: age, sex, BMI, creatinine, and medical history of hyperlipidemia, hypertension, and diabetes mellitus

### Plasma Irisin Level and Clinical Outcomes in AS

During the whole follow-up period, 52 (17.7%) patients suffered from all-cause death, and 23 (7.8%) patients died due to cardiovascular events. Compared with the high irisin level group, the all-cause death rate at the whole follow-up was significantly higher in patients with lower plasma irisin levels (23.1% versus 12.3%, *P* = 0.016). However, cardiovascular mortality at the whole follow-up was comparable in the two groups. Similarly, no significant difference in all-cause or cardiovascular mortality at the 1-month or the 1-year follow-up was noted between plasma irisin dichotomous groups. To further confirm the association between plasma irisin levels and all-cause mortality in AS patients, we conducted logistic regression analysis. Univariate logistic analysis indicated that all-cause mortality at the whole follow-up was significantly different between irisin dichotomous groups (*OR* = 0.467, 95% CI 0.250–0.873, *P* = 0.017). After adjusting for age, sex, BMI, creatinine, PAS or MAVD, and the calcification grade of the aortic valve in multivariate logistic analysis, the all-cause mortality of AS patients at the whole follow-up was no longer significantly different between the two groups (*OR* = 0.591, 95% *CI* 0.287–1.217, *P* = 0.153). The clinical outcomes of the dichotomous plasma irisin groups are shown in Table 5.

**Table 5:** Mortality rates of aortic stenosis patients after TAVI

	Low irisin level (*n* = 147)	High irisin level (*n* = 146)	*P*-value
1-month			
All-cause	2.0% (3/147)	2.1% (3/146)	0.993
Cardiovascular	1.4% (2/147)	2.1% (3/146)	0.646
1-year			
All-cause	6.8% (10/147)	6.8% (10/146)	0.987
Cardiovascular	5.4% (8/147)	4.1% (6/146)	0.593
Whole follow-up			
All-cause	23.1% (34/147)	12.3% (18/146)	0.016
Cardiovascular	10.2% (15/147)	5.5% (8/146)	0.133

**Fig. 3:**
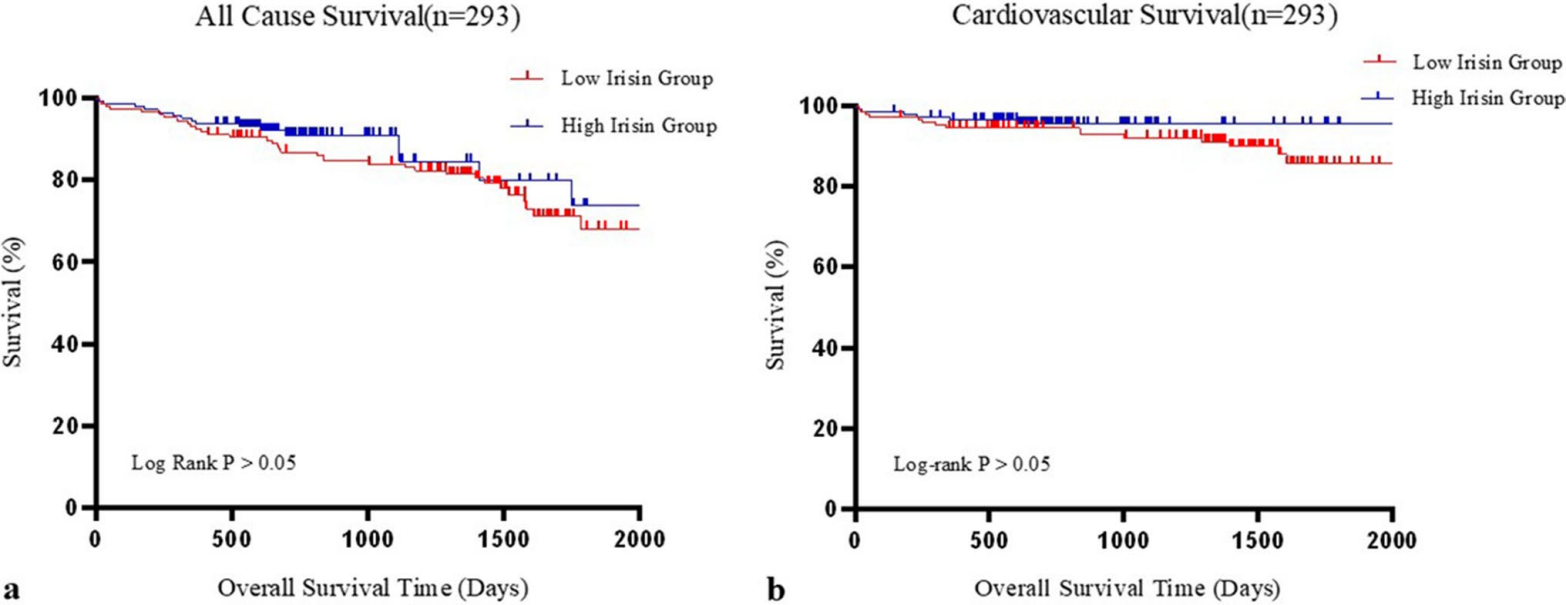
All cause (**a**) and cardiovascular survival (**b**) curves of aortic stenosis patients with different plasma irisin levels

To compare the time-to-event curve between different plasma irisin levels, Kaplan–Meier survival analysis with the log-rank test of all-cause and cardiovascular mortality was performed among all patients. The results indicated similar death rates of all-cause mortality between patients in the dichotomous irisin group throughout the entire follow-up (log-rank *P* = 0.507). Similarly, cardiovascular mortality rates exhibited no significant difference between the dichotomous irisin groups (log-rank *P* = 0.545) (Fig. 3).

## Discussion

Our study was the first to pay close attention to exploring the association of plasma irisin with baseline characteristics in AS patients and evaluating the implication of baseline plasma irisin levels on post-TAVI clinical outcomes of AS patients. Spearman rank correlation analysis indicated that frailty, PAS or MAVD, and the calcification grade of the aortic valve were strongly associated with plasma irisin levels. Further analysis demonstrated that the low plasma irisin level was independently associated with frailty, and the high plasma irisin level was independently associated with PAS. ROC curve analysis showed a significant predictive value of plasma irisin levels for PAS (*AUC* = 0.647, 95% *CI* 0.582–0.711, *P* < 0.001). Participants with massive aortic valve calcification had lower plasma irisin levels than patients with no aortic valve calcification. Plasma irisin levels were inversely associated with all-cause death rates during the whole follow-up period, whereas this association disappeared after adjusting for confounding variables. Additionally, survival analysis demonstrated similar time-to-event curves between patients in the dichotomous irisin groups during follow-up. We also explored the associations of other risk factors including pro-BNP, frailty scoring, and troponin with clinical outcomes (see supplemental materials).

Previous studies have demonstrated that cardiac muscles are capable of producing a large amount of irisin [9]. However, the role of irisin in the pathogenesis of cardiovascular diseases remains controversial. In the heart tissue and circulation of the murine MI model, irisin expression was lower and negatively correlated with cardiac damage markers, such as troponin and CK-MB [19]. Similarly, circulating irisin levels were decreased in patients with either stable coronary artery disease or MI versus healthy controls [12]. Researchers hypothesized that to resist reduced energy availability, the myocardium may release less irisin to restrain the metabolic demands in the state of deficient blood and oxygen supply [12]. According to this hypothesis, we suppose prolonged left ventricular ejection time and decreased cardiac output caused by AS results in myocardial ischemia and increased myocardial oxygen consumption, which subsequently leads to decreased irisin level in the heart tissue and circulation of AS patients. Shen et al. provided evidence that plasma irisin levels were significantly higher in AHF patients who died at the 1-year follow-up [14]. Increased irisin expression in hypertrophic murine heart and plasma improved cardiac function and reduced pressure overload-induced cardiac hypertrophy and fibrosis [20]. Therefore, we hypothesized that irisin expression may temporarily and reflexively increase as a compensatory mechanism when severe AS patients suffer acute heart failure or stress. Once severe AS is improved after TAVI, cardiac irisin expression levels may change. In addition, decreased activity tolerance caused by frailty in severe AS patients could reduce circulating irisin secreted by skeletal muscle, which may also change the intrinsic irisin balance of the body and influence the prognostic value of irisin. Therefore, baseline plasma irisin levels measured before TAVI may not strongly predict the prognosis of severe AS patients. Hospitalized patients we included had already been in a state of cardiac stress and heart failure due to severe AS, so further studies could consider measuring circulating irisin levels in AS patients with no symptoms or mild symptoms to exclude the influence of heart failure and cardiac stress state. In addition, we can also explore the relationship between irisin and AS by measuring postoperative circulating irisin levels before discharge as a baseline and monitoring changes in circulating irisin levels during postoperative follow-up. As a metabolically related biomarker, irisin may better reflect body status, which could replace subjective biomarkers such as frailty in risk evaluation and stratification of AS.

Previous studies demonstrated that patients with PAS who underwent TAVR had worse survival than patients with MAVD [21–23]. In addition, PAS without regurgitation is a risk factor for early stroke after TAVR [24]. However, patients with MAVD exhibited more frequent prosthetic valve regurgitation, a higher operative risk, more severe adverse cardiac remodeling, and worse functional status than patients with PAS [25]. Our study demonstrates that the plasma irisin level has a strong predictive value for PAS, suggesting that irisin could aid in the risk stratification of interventional therapy and prognosis evaluation for AS patients.

Calcific aortic stenosis is the most common cardiac valve lesion in developed countries [26]. The process of cardiac valvular calcification shares a similar pathophysiological mechanism with bone formation including promotion of osteogenesis and loss of mineralization inhibitors, which lead to the deposition of extracellular matrix and calcium phosphate crystals in cardiac valves [27]. Colaianni et al. indicated that irisin could induce osteoblast differentiation: increase the expression of osteoblastic transcription regulators, such as Runt-related transcription factor-2 and osterix; and upregulate osteoblast differentiation markers, including alkaline phosphatase, collagen type 1 alpha-1, osteocalcin, and osteopontin [28]. Consistent with our findings, a previous study also demonstrated that the irisin level was significantly lower in patients with vascular calcification than in those without vascular calcification. A lower irisin level was an independent risk factor for vascular calcification in hemodialysis patients [29]. More studies are needed to elucidate the pathophysiological mechanisms of the interaction between irisin and valve calcification in AS. It has been demonstrated that aortic valve calcification is significantly associated with an increase in LA dilation, left ventricular hypertrophy, peak velocity, and the mean gradient, and a decrease in AVA [30]. However, our study did not identify an association between circulating irisin and these hemodynamic parameters of AS severity. A large portion of our patients had AS combined with AR. Hemodynamic parameters of AS, such as valve diameter and peak velocity, are influenced by anatomical structure and systemic circulation pressure, especially when combined with AR. Therefore, low flow and a low gradient of the aortic valve caused by AR and decreased myocardial contractility would largely influence circulating irisin levels. The peak velocity did not accordingly increase in severe aortic valve stenosis with low flow and a low gradient, which would influence the study result. Therefore, we hypothesize that irisin may have a greater effect on metabolism than hemodynamic parameters.

It is well described that men present more calcification than women when assessing the physiopathology of AS in patients with the same AS severity [31]. Although our study also showed sex differences in aortic valve calcification, no sex difference in irisin levels was observed. Similarly, a cross-sectional study indicated no sex-specific difference in circulating irisin levels in a healthy cohort aged between 20 and 80 years old [32]. However, our study found that elderly men have lower irisin levels than elderly women. An animal study showed that the removal of sex hormones in ovariectomized rats resulted in increased circulating irisin [33], suggesting that a compensatory mechanism may exist in postmenopausal women.

The study was subjected to several limitations as follows. First, our analysis was a retrospective follow-up study. We only measured plasma irisin levels at baseline, which may not accurately reflect their distribution and change during the follow-up period. Second, this study was restricted to 293 AS patients who underwent TAVI in one institution without randomization and healthy population controls. Although we adjusted the outcome for diverse parameters, potential residual confounding factors may confuse the results. Undoubtedly, our findings need to be validated in a larger population and multiple institutions.

## Conclusion

Plasma irisin levels are negatively associated with the severity of aortic valve calcification, and high plasma irisin levels are an independent predictor for pure aortic stenosis in the context of mixed aortic valve disease. These findings imply an important role of irisin in aortic stenosis.

## Supplementary Information

Below is the link to the electronic supplementary material.

Supplementary file1 (DOCX 15 KB)

## Data Availability

The datasets generated during and/or analyzed during the current study are available from the corresponding author on reasonable request.

## Abbreviations

AS: Aortic stenosis
TAVI: Transcatheter aortic valve implantation
TAVR: Transcatheter aortic valve replacement
PAS: Pure aortic stenosis
MAVD: Mixed aortic valve disease
STS: The Society of Thoracic Surgeons
EuroSCORE: The European System for Cardiac Operative Risk Evaluation
FNDC5: Fibronectin type III domain containing protein 5
MI: Myocardial infarction
CK-MB: Creatine phosphokinase-myocardial band isoenzyme
AHF: Acute heart failure
CT: Computed tomography
VARC: Valve Academic Research Consortium
ELISA: Enzyme-linked immunosorbent assay
BMI: Body mass index
DM: Diabetes mellitus
PVD: Peripheral vascular disease
PCI: Percutaneous coronary intervention
eGFR: Estimated glomerular filtration rate
pro-BNP: Pro-brain natriuretic peptide
cTnT: Cardiac troponin-T
LVEF: Left ventricular ejection fraction
LA: Left atrium
PASP: Pulmonary artery systolic pressure
LVEDd: Left ventricular end-diastolic dimension
mPG: Mean pressure gradient
AVA: Aortic valve area
V_max_: Maximum transaortic velocity
AR: Aortic regurgitation
MS: Mitral stenosis
MR: Mitral regurgitation
TR: Tricuspid regurgitation
BAV: Bicuspid aortic valve
TAV: Tricuspid aortic valve
NYHA: New York Heart Association
IQR: Interquartile range
ROC: Receiver operating characteristic
IL-6: Interleukin-6
