# Supplementary material for: Circulating Irisin Level as a Biomarker for Pure Aortic Stenosis and Aortic Valve Calcification
Source: J Cardiovasc Transl Res. 2022 Oct 12;16(2):443–52. doi: 10.1007/s12265-022-10327-9 (PMC10151307; doi:10.1007/s12265-022-10327-9)
Supplement: Supplementary file 1 — Supplementary file1 (DOCX 15 KB) [file 12265_2022_10327_MOESM1_ESM.docx]

Supplementary Information

**SUPPLEMENTAL MATERIALS**

We exerted logistic regression analysis to explore the associations of other risk factors including pro-BNP, frailty scoring, and troponin with clinical outcomes. Univariate logistic analysis indicated that pro-BNP levels were significantly elevated in AS patients who suffered from all-cause death (OR=3.185, 95% CI 1.769-5.737, P<0.001) or cardiovascular death (OR=3.204, 95% CI 1.399-7.338, P=0.006). After adjusting age, sex, and creatinine, pro-BNP levels remained significantly associated with all-cause mortality(OR=2.429, 95% CI 1.302-4.533, P=0.005) and cardiovascular death (OR=3.026, 95% CI 1.292-7.086, P=0.011). Univariate logistic analysis showed that frailty was associated with all-cause mortality in AS patients (OR=3.771, 95% CI 1.439-9.882, P=0.007). Further multivariate logistic regression analysis revealed that frailty was independently associated with all-cause mortality when adjusting for age, sex, and creatinine level (OR=3.453, 95% CI 1.293-9.222, P=0.013). However, troponin was not associated with either all-cause mortality or cardiovascular death in this cohort of AS patients.
